# Vulnerability as a normative concept: reviving the scepticism

**DOI:** 10.1007/s11019-026-10345-3

**Published:** 2026-04-04

**Authors:** Hojjat Soofi

**Affiliations:** Sydney Health Ethics, Sydney School of Public Health, The University of Sydney, Edward Ford Building A27, Sydney, NSW 2006 Australia

**Keywords:** Vulnerability, Obligation, Normative concepts, Eliminativism, Needs, Dependence

## Abstract

Appeals to vulnerability are widespread in bioethics, yet they continue to face criticism. One line of critique concerns their normative force, with two major sceptical claims dominating the debate. The strong version holds that vulnerability alone carries little or no normative force. The moderate version grants some role but denies that it has the same normative force as concepts such as wrong or wronging. I revisit these claims in light of Catriona Mackenzie’s rejoinder, which shifts the focus from relational or situational vulnerability to its ontological sense, framed as a morally salient feature of humanity. While Mackenzie’s account is novel, it faces challenges from the needs‑theory literature she draws on, where dependence appears a more direct basis for grounding obligations related to vital needs. I examine Mackenzie’s favoured argumentative strategy of intertwining dependence and ontological vulnerability, and I argue that even if one accepts intertwinement in functional co‑constitution sense, this would not by itself establish vulnerability as a foundational obligation‑grounding concept. I then consider possible avenues for vulnerability defenders. I argue that the option of identifying another transitive intra‑conceptual relation between dependence and vulnerability is not readily available. A second option is to claim that ontological vulnerability is foundational not through a transitive relation from dependence but because both are morally salient. I argue that the stronger sense of moral salience implies obligation‑grounding, making the argument circular; and that the weaker sense, understood as mere moral relevance, leaves the account open to the very sceptical challenge it aims to resist.

## Introduction

Vulnerability is a commonly cited concept in bioethical literature (Mergen and Akpınar 2021; ten Have 2016). The concept is thought to be “an important concept in bioethics” (Rogers 2013, p. 60) and “[c]oncern for human vulnerability seems to be at the heart of bioethical inquiry” (Rogers et al., 2012).

But the vulnerability talk in bioethics has also faced criticism. One line of criticism concerns the normative force of appeals to vulnerability, which, as Catriona Mackenzie notes, “is often simply assumed rather than explained” (2016, p. 83). Mackenzie’s example is the reaction to Australia’s 2014 federal budget, widely criticised for targeting “the health and life opportunities of the most vulnerable sections of the Australian community” (Parliament of Australia, 2014). In the post‑budget debate, vulnerability talk dominated, and, as Mackenzie notes, it was treated as self‑evident that certain forms of (increased) vulnerability generate obligations of justice. In this usage, vulnerability is assumed to be normatively substantive in the sense that the budget was deemed unjust because it would increase vulnerability, and that fact *alone* was taken to demonstrate a failure to meet certain duties of justice (and to justify certain duties of remediation). It matters to be clear whether this assumption is true, because if it is not (and vulnerability is just, say, descriptive or rhetorical marker), we risk misdirecting our deliberative efforts and potentially overlooking more fundamental grounds of obligations.

This kind of assumption is what scepticism about the normative force of appeals to vulnerability calls into question. In the extant bioethics literature, two major forms of such scepticism have been raised, and their key claims can be formulated as follows:

*Strong Sceptical Claim* or *SSC*: appeals to vulnerability, on their own, do not carry any (significant) normative force.

*Moderate Sceptical Claim* or *MSC*: appeals to vulnerability, on their own, do not carry the (significant) normative force that appeals to concepts such as wrong or wronging carry.

Forms of SSC are at play, for instance, in the eliminativist approach to vulnerability as favoured by Wrigley (2015) and Wrigley and Dawson (2016). According to this approach, appeals to vulnerability can be challenged on the grounds that they are “unnecessary” (Wrigley 2015, p. 486) or “redundant” (Wrigley and Dawson 2016, p. 208) as such appeals do not serve a substantial normative function. In other words, the normative force that appeals to vulnerability seem to carry may be traced, upon analysis, to other moral considerations and/or underpinning moral theories (Wrigley 2015; Wrigley and Dawson 2016). The upshot is, then, that we can and should do away with vulnerability as a substantive moral concept.

MSC is, for instance, at play in the deflationary approach to vulnerability as proposed by Samia Hurst (2008). This approach does not call for eliminating vulnerability from the list of substantive moral concepts one can rely on. Instead, the approach aims to offer (or gesture at the possibility of offering) a definition of vulnerability that makes it explicit that the normative force of vulnerability-related claims to (special) protection, “does not rest with vulnerability itself” but rests with other moral concepts whose normative force is stronger and arguably less disputed e.g., as Hurst suggests, the concepts of wrong and wronging (Hurst 2008, p. 191).

My goal in this paper is to revisit and reexamine SSC and MSC in light of the rejoinder offered by Catriona MacKenzie (2016), which remains the most direct and explicit attempt to address the core concerns about the normative force of appeals to vulnerability. In doing so, and with the aim of linking SSC and MSC more directly to Mackenzie’s defence, I narrow my focus and take the direction of the sceptical arguments in the following ways.

First, my primary focus here will be on different *conceptions* of vulnerability as deployed or featured in appeals to vulnerability and not on different concepts of vulnerability. Here, following Rawls (1999), I make a distinction between concepts and conceptions.^1^ I take concepts to be broad terms, the specifics of which can be delineated by different conceptions. So, there may be different concepts of vulnerability such as epistemic vulnerability of the sort discussed by Casey Johnson (2020), vulnerability as an explanatory concept such as the one examined by Wrigley (2015), vulnerability as a descriptive or factual designator, or vulnerability as a normative concept. I will be concerned here with different conceptions of vulnerability construed as a *normative* concept.

Second, and relatedly, the normative function of invoking vulnerability that will be my focus is the manner in which such invocations (are presumed to) trigger, provide the foundation for, or capture particular (vulnerability-related) inter-personal moral obligations. More specifically, using Darwall’s framing (2013, ch. 2), my focus, will be on putative obligors (those subject to obligations) and not on obligees (those to whom obligations are owed). In this respect, my analysis takes a different argumentative direction than the one taken in Hurst’s analysis, where the focus is primarily on (obligees’) claims to “special protection”^2^.

Third, I concentrate on one specific aspect of eliminativist reasoning: the critique of vulnerability’s normative role. Wrigley’s (2015) broader eliminativist argument, for instance, is motivated by concerns about explanatory adequacy and the difficulty of defining necessary and sufficient conditions for the general application of vulnerability, especially when vulnerability is invoked both as a universal feature of human life and as a distinguishing feature of specific sub-populations. While the critique concerning the extension of the concept motivates a move to eliminate (the concept of) vulnerability from normative deliberation, I take it that this normative eliminativism is not exclusively dependent on the critique of vulnerability’s conceptual scope. That is, even if there are good reasons to think that the extension of vulnerability is no more indeterminate than that of other normative concepts such as autonomy or harm, which would address some concerns about the conceptual scope of vulnerability, the eliminativist challenge to the normative function of vulnerability remains a live and pressing issue.

Here is how the paper is structured.

Section Introduction reconstructs SSC and MSC along the directions specified above. I do this by focusing on two types of conceptions of vulnerability: interest-based and consent-based. I focus on these conceptions as they more clearly reveal the force of the criticisms. They also have certain advantages, so I would not target implausible or easily dismissible accounts. Another reason is that interest-based and consent-based conceptions seem to capture the core idea behind many other conceptions of vulnerability deployed in the extant literature. For example, the exploitation-centred conception cited by Hurst (2008) can be seen as a variant of the consent-based view. Finally, discussing these two conceptions helps highlight the distinctiveness of MacKenzie’s (2016) approach. Against this background, Sect. Reconstructing SSC and MSC presents Mackenzie’s response to the sceptical claims. Section Mackenzie’s defence critically examines her account. I raise a challenge drawn from the needs-theory scholarship she relies on, which suggests that dependency, rather than vulnerability, provides a more fitting basis for grounding obligations related to vital needs. I then consider and raise doubts about the strategy that the intertwinement between dependence and ontological vulnerability can address this challenge. Next, I explore an alternative reading: that both dependence and ontological vulnerability are morally salient features of humanity and thus foundational. I argue that this appeal risks circularity or relegates vulnerability to a secondary role if moral salience is understood merely as moral relevance. The paper concludes with a summary and a brief discussion of the implications for the debate.

## Reconstructing SSC and MSC

To reiterate, my aim in this section is to demonstrate the force of the sceptical claims by focusing on two familiar strands of thinking about vulnerability.

Here is one formulation of the first familiar thought:

*B*^3^*has a moral obligation to do ɸ for*,* or to adopt attitude ψ towards*,* A if*,* and because*,* A is vulnerable to B’s actions or attitudes due to a diminished capacity to give informed consent and/or a lack of access to the material or relational conditions required for such consent.*

Let us call the above a consent-based conception of vulnerability (CBCV). It is a more expanded and qualified version of, for example, what Hurst describes as “consent-based vulnerability” (2008, p. 192)^4^ or of the view expressed in a Nuffield Council on Bioethics (2007) report (titled ‘Public health: ethical issues’), which invoked vulnerability as “lacking capacity to make informed judgements for oneself, being socially or economically disadvantaged, or … the result of other factors that contribute to a lack of autonomy.” (p. 144)

CBCV has some appealing features.

First, it is appropriately focused on the normative dimension of appeals to vulnerability. Second, it does not narrow the scope of vulnerability-related obligation to actions: it creates a conceptual space for the (potentially useful but not so widely examined) idea that vulnerability-related obligations are not solely about taking certain actions^5^ but there are also about adopting certain (suitably defined) attitudes.^6^ Third, nothing in the above formulation of CBCV commits us to the view that there are certain paradigmatic vulnerable (sub-)populations. Taking this view, as, e.g., Rogers (2013), Luna (2009, 2019), Luna and Vanderpoel (2013) and Levine et al. (2004) note, would lead to the problematic labelling or stereotyping of some groups of population (e.g., “vulnerable older adults living with advanced dementia”) or to avoid the problematic implication that, as Fineman puts it, “[t]hose who stand outside of the constructed vulnerability populations are thus fabricated as invulnerable” (2013, p. 16). And, fourth, the kind of moral obligation that the above formulation of CBCV refers to is not just to those whose capacity for consent is diminished but also to those who may have the capacity but may not have access to (sufficient) material resources (e.g., accessible information) or relational resources (e.g., supportive family or community members).

So, in short, CBCV seems to be aptly focused, conceptually expansive, not leading to labelling or stereotyping, and captures a wide range of consent-related moral obligations.

But, CBCV has a significant shortcoming. The shortcoming is that it is consent not vulnerability that, in CBCV, does the primary (if not all) normative work. CBCV reference to “vulnerable to”, when considered in isolation, does not seem to have much direct bearing on the kind or arguably even the weight of the moral obligations that CBCV encapsulates. In short, what CBCV encapsulates appear to be consent-based obligations not vulnerability-based obligations.

Note that this does not mean that invoking vulnerability in CBCV does not do *any* normative work. The point is that CBCV does not, normatively speaking, rely much on vulnerability per se to specify the moral obligations at stake. Reference to vulnerability in CBCV (in “vulnerable to”) seems to function mainly, drawing on Wrigley, like a “linguistic device” to direct attention to those who are at risk of being negatively impacted (by B) (2015, p. 478). So, in this sense, reference to vulnerability in CBCV does some (arguably minimal) normative work but not the kind of normative work that would make CBCV much resistant to the criticism motivating SSC and MSC. Alternatively stated, it would make little (according MSC) or no (according to SSC) normative difference if we did away with vulnerability in CBCV and replaced it with, say, at risk of being negatively impacted 7.

I now move on to consider another familiar strand of thinking about vulnerability. Here is one formulation of it:

*B has a moral obligation to do ɸ for or take attitude ψ towards A if*,* and because*,* A’s interest is vulnerable to be set back by actions or attitudes of B*.

Let us call the above an interest-based conception of vulnerability (IBCV)^8^.

In some respects, IBCV is akin to an extant conception of vulnerability as deployed by Robert Goodin (1985) in that IBCV similarly makes conceptual links between vulnerability and interest. There are, however, some differences that will become clear later.

But before that, it is apposite to discuss some of the merits of IBCV. Similar to CBCV, IBCV is clearly focused on the normative dimension of appeals to vulnerability, focuses both on actions and attitudes, and avoids problematic labelling or stereotyping of some groups of population.

IBCV, however, is normatively under-specified. Its reference to (A’s) interest is excessively broad. It is compatible with IBCV to say that B has some moral obligations in relation to A if B’s actions or attitudes sets back *any* interest of A.

One way to defend IBCV from the above charge is to qualify the scope of interests under consideration. This is what Goodin’s (1985) conception expressly does. Unlike IBCV, Goodin’s conception narrows the scope of interests to what he calls “vital interests” (1985, p. 111). 9 These are interests that, according to Goodin, need to be protected as a matter of providing “primary goods” to people (1985, p. 111). For Goodin, some of the possible candidates for being considered vital interest are food, clothing, and shelter.

Goodin, however, takes a step further. He links his version of IBCV to a background moral theory. This move is meant to provide, among other things, an explicit justificatory story for *why* B has a moral obligation in relation to A‘s (vital) interests (suitably defined). Goodin’s preferred moral theory is a form of welfare-consequentialism. There is a consequentialist moral principle,^10^ Goodin argues, that enjoins us “to produce certain sorts of consequences … that protect the interests of those who are particularly vulnerable to” our actions and choices (1985, p. 114). Goodin, then, adds that this requires us to give some form of special consideration to those who are especially vulnerable to our actions and choices not in the sense of giving extra weight to their interests but, instead, in the sense of directing attention to specific opportunities we have to impact their interests more significantly than others (through our actions and choices).

Goodin has a lot more to say about vulnerability-related model of moral obligations. But the above suffices to see how Goodin’s model seems to have more normative content and specification than the initial formulation of IBCV mentioned earlier.

Despite such helpful features, Goodin’s conception of vulnerability still can be challenged by the sceptical arguments under consideration. This is because vulnerability for Goodin seems to be merely^11^ a descriptive concept to refer to certain forms of susceptibility to be negatively impacted by others. The main normative work in Goodin’s model is done by the concept of vital interests and the underpinning moral theory he deploys. As Panitch and Horne note, we can say that in Goodin’s model, vulnerability functions as “a mere middleman” (Panitch and Horne 2016, p. 102). A middleman that may have some descriptive utility or useful rhetorical force to, e.g., help identify to whom we are morally obligated, but it does not (or as Panitch and Horne argue, cannot) normatively ground the (type of) moral obligations Goodin refers to.

To be clear, the claim made above is not that Goodin’s (1985) model of moral obligations does not do the expected normative work. Rather, the claim is that the conception of vulnerability that Goodin deploys in his model of moral obligations, on its own, is largely normatively inert (i.e., in terms of normatively grounding the obligations). To reiterate, it is the concept of vital interests and the underpinning moral theory, not vulnerability, that Goodin relies on to *ground* our moral obligation to meet others’ vital interests.

In summary, the force of SSC and MSC can be seen in relation to two familiar strands of thinking about vulnerability, namely, the consent-based and interest-based approaches as formulated above. In both cases, it is not vulnerability that grounds or specifies the moral obligations. Rather, the moral obligations at stake are grounded or specified by other concepts such as consent and vital interests or by an underpinning moral theory such as welfare-consequentialism. The upshot of the sceptical arguments would be that replacing vulnerability with something like “susceptible to or at risk of being negatively impacted” make little or no normative difference.

## Mackenzie’s defence

I now turn to Mackenzie’s response to the sceptical claims questioning whether vulnerability functions as a normative concept.

MacKenzie (2016) begins from a different starting point than CBCV and IBCV, which themselves differ in important respects but share an emphasis on deploying vulnerability in a “relational and context-specific” sense (p. 87), that is, as being vulnerable *to* something or within a particular *context*. She draws on the idea that what grounds our moral obligations to other human beings are what we take to be “the morally salient features of humanity that ought to guide our deliberations and actions” (2016, p. 91). One of these morally salient features, according to Mackenzie, is our *ontological vulnerability*. She is, nonetheless, explicit that ontological vulnerability is not the only morally salient feature of humanity. There are other features such as (but not limited to) sentience, our rational/agential capacities, and neediness.

Mackenzie, in particular, emphasises the intertwining of neediness and vulnerability. The intertwinement, for Mackenzie, manifests in two ways.

First, she holds that vulnerability is causally linked to neediness: “we have needs because we are vulnerable …” (2016, p. 94). Second, our neediness and vulnerability intersect in giving rise to moral obligations: on her account, our shared humanity and interdependence ground obligations to ameliorate vulnerability and meet the vital needs of others.

In explaining what is meant by vital needs, Mackenzie draws on Miller (2013). On Miller’s account, vital needs refer to those needs that, if unmet, will lead to serious agency-undermining harms. A clear example of such needs, for Miller, is the need to receive sufficient social recognition. In meeting the vital needs, Miller holds, we are dependent on others and are vulnerable to the impact of their actions/attitudes on us throughout our lives. As such, we cannot will indifference toward meeting the vital needs of those dependent on us without risking contradiction (in will). For Miller, this means that, In Kantian terms, we have an imperfect duty of beneficence (or care) to attend to the vital needs of those who are dependent on us.^12^

Another premise of MacKenzie’s (2016) argument is that the vital needs fall under the category of non-instrumental needs. On this point, following Wiggins (1998; 2024), MacKenzie (2016) draws a distinction between instrumental needs and non-instrumental needs.^13^ Instrumental needs are stipulated as those (types of) needs that have the following underlying conceptual structure: X needs Ys in order to realise ends Zs. Instrumental needs (Ys) are, then, taken to refer to necessary conditions for X realising ends Zs.

Contrarily, non-instrumental needs have a different underlying conceptual structure: X needs Ys if (and only if) X is to avoid states Ws. Further, in some relevant respect, our very reference to X entails the necessity of X avoiding state Ws.^14^

In this latter sense, satisfying needs Ys, when Ys are non-instrumental *and* vital, would be normatively significant not because meeting Ys realises certain ends but because the kind of entity that X is, in some relevant moral respects, logically or definitionally entails the necessity of meeting Ys. To use the above-mentioned example of the need to receive sufficient social recognition, one might say that that need is non-instrumentally vital in the sense that we *qua* moral agents (or persons), by stipulation, are the kind of entity that will be seriously harmed or will fail to flourish if we do not receive sufficient social recognition. Alternatively stated, using Wiggins’s formulation (1998, p. 10), moral agents (or persons) are the kinds of entities that, by stipulation, if they are to avoid being seriously harmed or to avoid not being able to flourish, then they necessarily should have sufficient social recognition.

## Challenging Mackenzie’s defence

In what follows, I examine a challenge, which arises from the very scholarship Mackenzie draws on to develop her response.

Mackenzie’s strategy is to defend ontological vulnerability as a normative concept by linking it to vital needs, drawing on Miller’s (2013) account of those needs. Yet Miller is critical of treating vulnerability as a foundation for moral responsibility. Although their critique targets responsibility rather than obligation, it nevertheless signals a transposable worry about the priority structure in our thinking about moral obligations related to vital needs. The concern, in Miller’s own words is this:

… Why is vulnerability ill-suited to be the foundation of moral responsibility?

In short, protection in light of our vulnerability is not all that we require … When protection in the face of vulnerability fails and we are injured and when we are needy because no one is there to meet those needs, we occupy the same position – one of dependency. The most foundational component of our human existence is not our vulnerability, but rather, our dependency. (2013, pp. 658-9)

As I see it, the key insight is that vulnerability and vital needs operate in two different, albeit related, conceptual registers.

The thought seems to be that vulnerability (both in relational and ontological senses) operates within the register of possibility: it marks agents’ risk of *exposure* to, for example, harm or deprivation (as when a patient with severe asthma is vulnerable to a fatal attack if treatment is delayed). By contrast, vital needs are concepts in the register of necessity (as when that same patient, during an acute attack, requires immediate oxygen to survive). Accordingly, the kind of normative response that appeals to vulnerability can attract would be forms of protection, such as shielding individuals from harm. The normative response to vital needs, however, takes a different form: while it may include protection, it should ultimately be oriented toward the *provision* of concrete material or relational support. This type of support can only be provided within caring relationships embedded in background dependency (or interdependence). So, the upshot would be that dependency seems a more fitting concept for explaining the ground of moral obligations than vulnerability as far as the obligations to meet others’ vital needs are concerned.

To be fair, Mackenzie explicitly describes vulnerability and dependency as “intertwined” (2016, p. 85). This seems (to me) to support the thought that even if the conceptual connection between dependence and vital needs is more straightforward as Miller suggests or, put differently, even if dependence and vital needs operate on the same conceptual register in a way vulnerability initially appears not to, the intertwining relation matters. That is, if dependence serves as a foundation for moral obligations (related to vital need), and vulnerability is conceptually bound up with dependence, then the grounding role attributed to dependence extends to vulnerability as well. The resulting picture is a modest *transitivity* claim: dependence grounds moral obligation, dependence and vulnerability are intertwined, and thus vulnerability shares that foundational status.

On the face of it, however, two concepts can be tightly connected without both necessarily grounding moral obligation. So, even if one of them is foundational, it is not immediately clear that their intertwinement alone entails that the other must be as well.

To unpack this further, I examine a sense in which the concepts of vulnerability and dependency might be thought to intertwine, namely, functional co-constitution. My aim is not to defend this account, but to show that even if we accept this sense, it would not directly determine which concept or more broadly what is foundational to moral obligations related to vital needs.

Functional co-constitution can be thought of as a relation in which two concepts are deployed in ways that partly determine the extension and explanatory role of each other. The point is not merely that the concepts frequently appear or are cited together, but that the functional role of each concept is partly realised by drawing on features or facts that determine what falls under the other concept. That is, in deploying one concept, we rely on elements that help fix the extension of the other, so that each concept’s practical application is determined by the other’s constitutive boundaries^15^.

For example, residents in aged care facilities are commonly considered vulnerable (Sanchini et al., 2022). This is due to their susceptibility to harm in virtue of certain dependencies, such as reliance on staff for daily care or on institutional systems for safety and support. Conversely, in specifying what counts as a dependence, we often appeal to facts about how these relationships expose residents to distinctive forms of vulnerability through neglect, abuse, or restricted autonomy. In this sense, the concepts are co-constituting: the extension of each is partly fixed by its relation to the other in terms of their usage.

But even if we accept this functional, co-constitutive relation, it applies more straightforwardly to *situational* or *relational* vulnerability than to ontological vulnerability. Ontological vulnerability, by stipulation, does not *depend* on particular relational structures: it is a universal, background condition of human life arising from “our common embodied humanity and equal susceptibility to suffering” (Mackenzie, p. 85).

There is, however, a live option to extend the functional co-constitution story to ontological vulnerability. One could argue that every situational or relational manifestation of vulnerability is connected to, or underpinned by, universal ontological vulnerability, that is, the latter reverberates through all instances of relational vulnerability. This aligns with Mackenzie’s attempt to “reconcile” the relational/context-dependent and universal/ontological dimensions of vulnerability (p. 97). In her account, inherent vulnerability arises from features intrinsic to the human condition while situational vulnerability emerges from context-specific factors such as social, political, or environmental conditions. The idea is that although conceptually distinct, these sources are mutually implicated: situational vulnerabilities both instantiate and interact with underlying inherent vulnerabilities, and the effects of the latter are mediated through relational and social structures, including care practices and institutions.

I remain agnostic as to whether this clearly establishes that the functional co-constitution story applies to ontological vulnerability, but even if we grant such an intra-conceptual relation between ontological vulnerability and dependence, it does not follow that if one concept is foundational to moral obligation, the other must be as well. Arguing for that conclusion would require an additional, independent step, and it is far from obvious how one could move from the mere fact of intertwinement (in the above sense) to the following stronger claim:

*If the two concepts are functionally co-constitutive and one of them (C₁) is foundational to needs-related moral obligations*,* then the other (C₂) must also be foundational*^16^.

To clarify, I am not saying that the above logically rules out both ontological vulnerability and dependence being foundational to moral obligations (related to vital needs). My claim is rather conditional: even if one accepts the intertwining of these concepts in the sense of functional co-constitution, this does not by itself establish vulnerability as a foundational obligation-grounding concept.

Let me now canvass a few ways in which vulnerability theorists can address the above objection and advance the debate.

A possible strategy for vulnerability theorists is to posit another intra-conceptual relation (call it R) between dependence and vulnerability, such that if dependence is foundationally obligation-grounding, that status transfers to vulnerability in virtue of R. The difficulty is that no such transit-conferring relation is obvious. My hypothesis is that this difficulty arises, at least in part, from the said asymmetry between dependence and vulnerability in their respective conceptual registers. Any relation capable of doing the required transitive work would need to bridge the register of necessity, within which dependence and vital needs operate, and the register of possibility, within which vulnerability operates.

Of course, the above discussion is relevant if, as Mackenzie would likely hold, dependence *is* foundational to moral obligations concerning vital needs. To the best of my knowledge, there is no textual evidence suggesting otherwise. Indeed, Mackenzie explicitly adopts a pluralistic approach to morally salient features of human beings, and (inter)dependency appears to be one of these features (pp. 91 − 2).

In light of the above, a second possible argumentative strategy for vulnerability theorists is to abandon (or do not rely on) the assumption that the foundational status of ontological vulnerability *transits* from dependence. Instead, they could advance the claim that both are, in Mackenzie’s own terms, “morally salient characteristics of humanity” and, hence, each is foundational to obligations related to vital needs.

Mackenzie seems explicit about this in the following passage, though her focus is on vulnerability-needs pair rather than vulnerability-dependence pair:

*“…because vulnerability and needs are morally salient features of our humanity that ground obligations and ought to guide moral deliberation and action.”*(p. 97).

The success of this line of reasoning would depend on the sense of moral salience we employ. In what follows, I consider both a strong and a weak sense, and I show that neither is likely to offer much support to vulnerability theorists.

First, let us consider the strong sense of morally salient. In this sense, saying that X is a feature of being human would amount to saying that X *matters* for *grounding* moral obligations, duties, or responsibilities (or some other relevant notions). By deploying this sense of morally salient, however, we would risk circular reasoning. If we want to argue that ontological vulnerability is foundational to moral obligations and then we justify that by saying it is morally salient, we risk assuming what we are trying to establish. In other words, the argument risks going in a circle: the claim that vulnerability is morally salient already presupposes that it grounds obligations (or matters for grounding obligations). The real question, why and how ontological vulnerability generates obligations, still needs an (independent) answer.

By way of analogy, consider asserting that sentience is a morally salient feature of our shared humanity (in the abovementioned strong sense of salience), and then arguing that sentience should guide our deliberations as an obligation‑grounding concept. This reasoning would be similarly circular: the claim that sentience is morally salient already assumes the very point one is trying to establish, namely that it can serve as a basis for moral obligations.

Vulnerability theorists would avoid this problem of potential circular reasoning if they deploy a weaker sense of salience. It is conceivable that a feature X could be considered morally salient without, on its own, grounding obligations, provided that moral salience is understood simply as *relevance* for moral consideration (i.e., salience as relevance). On this view, an additional normative principle P would be needed to articulate obligations related to X in that, If X is morally salient and the normative principle P applies to X, then X grounds an obligation^17^.

This preserves the idea that features such as vulnerability matter morally, while locating the obligating force in the independent principle P. In doing so, it avoids circularity in appealing to moral salience itself. For example, the relevant normative principle P might be something like attending to the vital needs of others.

However, although this move avoids circularity, it invites a different worry: similar to consent-based and interest-based conception I discussed above, vulnerability no longer appears to be doing much of the normative work, since the obligation is now articulated primarily through appeal to P rather than through vulnerability itself. So, even under this weaker construal of moral salience (that is, salience as mere relevance), the argument would still fail to achieve its purported aim. Consequently, this strategy offers little argumentative support for vulnerability theorists who aim to defend (ontological) vulnerability as a substantive normative concept of the sort I have focused on.

## Conclusion

In this paper, I revisited two forms of scepticism about the normative force of appeals to vulnerability in bioethics. The first was eliminativist, claiming that vulnerability alone carries no significant normative weight. The second concedes some role but denies that vulnerability has the same force as concepts like wrong or wronging. To illustrate the force of the scepticism, I examined two familiar conceptions of vulnerability: interest-based and consent-based. Both are relational or situational (i.e., vulnerable to), and in neither case does vulnerability itself seem to ground obligations. Rather, obligations are articulated through appeals to other concepts such as consent or vital interests, or derived from broader theories like welfare consequentialism. The sceptical upshot is that replacing vulnerability with terms like ‘susceptible to’ or ‘at risk of harm’ would make little normative difference.

I then turned to Catriona Mackenzie’s response to these sceptical concerns. Her proposal shifts the focus from relational senses of vulnerability to its ontological sense. She argues that moral obligations are grounded in morally salient features of humanity, one of which is ontological vulnerability. A key assumption behind Mackenzie’s rejoinder is the intertwining of neediness and vulnerability: vulnerability is causally linked to neediness, and together they give rise to obligations to ameliorate vulnerability and meet vital needs.

However, Mackenzie’s account faced a challenge from the needs-theory scholarship she draws on. For Miller, dependency seems a more fitting basis for grounding moral obligations than vulnerability. This is because vulnerability and vital needs operate on different conceptual registers: the former in the register of possibility, the latter in the register of necessity. The normative response to vital needs, therefore, takes a different form. While it may include protection, it ultimately requires providing concrete material or relational support within caring relationships embedded in dependency or interdependence.

I then explored how Mackenzie’s rejoinder could address this challenge by asking whether, as she claims, there is an intertwinement between dependence and ontological vulnerability such that if the former is foundational to grounding moral obligation, the latter could be as well. I argued that this transitivity move would fail at least as long as intertwinement operates in something like a functional co-constitution sense. Even if there are good reasons to accept that ontological vulnerability and dependence are intertwined in this sense (which itself faces sceptical challenges), it is not clear that such intertwinement would be transitive in conferring foundational status from dependence to vulnerability.

I then considered two alternative argumentative strategies for vulnerability theorists.

The first is to identify another intra‑conceptual relation between dependence and vulnerability, such that if dependence is foundationally obligation‑grounding, that status transfers to vulnerability in virtue of that identified relation. While I cannot say that this strategy is doomed, it is not clear that any promising option is (at least readily) available.

The second strategy is to claim that the foundational status of ontological vulnerability does not derive from dependence; rather, both are, in Mackenzie’s own terms, “morally salient characteristics of humanity” and, hence, each is foundational to obligations related to vital needs. I considered two senses of moral salience and argued that neither would be of much help to vulnerability theorists. If moral salience is taken to imply being a ground for obligation or being something that matters for grounding obligation, then this risks circularity since saying something is morally salient would be akin to saying it matters for grounding obligations. If, alternatively, moral salience is taken to mean moral relevance, then it would render vulnerability talk primarily secondary to the moral obligation story as another step would be required in the inference, such as a normative principle that would then carry the primary normative work, which would be precisely what the sceptical concern targets.

Of course, vulnerability theorists can still pursue the second strategy by stipulating moral salience in alternative senses that would not force them to choose between circular reasoning and rendering vulnerability talk normatively secondary. But it is not clear to me how that can be a live option, given the seeming normative elements baked into the notion of moral salience.

Perhaps a better move for vulnerability theorists would be to address the sceptical concerns indirectly. One approach that I remain agnostic about, but that I think is worth exploring, is the emerging philosophical method of “conceptual reverse‑engineering” (O’Sullivan and Mace 2025; Queloz 2021). The starting point would be to analyse what would be lost if appeals to vulnerability were merely diagnostic, explanatory, rhetorical, or pragmatically useful (or were to remain in the background merely as anthropological descriptions or explanations). They could then establish that these losses matter in ways that require mitigation, and reverse‑engineer the concept of vulnerability so that it captures what is lost and supports the required mitigation.

## Footnotes

1 For a more expanded account of this distinction see Sessions (1981).

2 While Mackenzie’s account, as I discuss later, is relational, and while isolating one side of the obligor–obligee dynamic introduces a degree of abstraction, this focus is adopted deliberately in order to give the inquiry a clear and manageable scope, rather than to de-emphasise the relational dimension at stake. I thank the anonymous reviewer for drawing my attention to this point.

3 I refer to B as an individual here, but the point can be made, *mutatis mutandis*, about groups of individuals, too.

4 This emphasis on consent is also evident in the account of vulnerability that Kipnis (2001) offers in the research ethics context (though I think Kipnis’ account is best characterised as consent-focused rather than consent-based).

5 This is, however, a contested point. For an insightful discussion of whether there can(not) be attitude-centred obligations beyond (or somewhat non-derivatively from) action-centred obligations, see Clarke (2023).

6 Or negatively framed, CBCV creates a conceptual space for the idea that there are vulnerability-related obligations that may be contravened not only by failing to take certain actions but also by the failure to adopt certain attitudes.

7 An alternative way to frame this, following Formosa (2013), can be “at risk of being inflicted.”

8 It can also be called a harm-based conception if we replace ‘set back’ with ‘harmed’ in the above formulation. For simplicity’s sake, I use only one term.

9 Goodin (1985) references needs as well, but for brevity’s sake, I reserve a detailed examination of this term for later, choosing to focus solely on vital interests here. Also, it should be noted that Goodin does not suggest that we should opt for a fixed interpretation of interests.

10 Goodin (1985) frames this as “the principle of protecting the vulnerable.” He notes that this principle, while consequentialist in its form, aligns, in broad terms, with many non-consequentialist moral theories.

11 I acknowledge that this may be considered a rather strong claim. A weaker version of this claim can refer to ‘primarily’ instead of ‘merely’. The point that I make here, though, does not depend on the choice of framing.

12 Miller (2013) defends this line of reasoning against the back-scratching objection, according to which the argument from the imperfect duty of beneficence enjoins us to care for the present vital needs of others *mainly* to ensure that others will care for our vital needs in the future. See Miller (2013, pp. 57–58) for more on this.

13 I use the term non-instrumental needs for the sake of simplicity. MacKenzie (2016) refers to them as categorical and/or fundamental needs.

14 I should acknowledge that this is a rather truncated version of the longer formulation that Wiggins (1998) provides.

15 Here is a formal way to present the idea: *For concepts C₁ and C₂*,* if the application of C₁ (App(C₁)) depends on facts or properties that fix the extension of C₂ (Ext(C₂))*,* and the application of C₂ (App(C₂)) depends on facts or properties that fix the extension of C₁ (Ext(C₁))*,* then FuncCoCons(C₁*,* C₂) holds.*

16 Here is one way to present the idea in a formal schema: *If the two concepts are functionally co-constitutive (FuncCoCons(C₁*,* C₂)) and one of them (C₁) is foundational to needs-related moral obligations (F(C₁))*,* then the other (C₂) must also be foundational. That is*,* FuncCoCons(C₁*,* C₂) ∧ F(C₁) ◊ F(C₂).*

17 Formally, this can be presented as follows: *MorallySalient(X) ∧ P(X) → GroundsObligation(X)*.
